# Sero-survey on long-term care facility residents reveals increased risk of sub-optimal antibody response to BNT162b2: implications for breakthrough prevention

**DOI:** 10.1186/s12877-022-02884-0

**Published:** 2022-03-10

**Authors:** Barbara Caimi, Marco Franzetti, Rossella Velleca, Alessia Lai, Antonella Gatti, Pier Luigi Rossi, Marco D’Orso, Fabrizio Pregliasco, Claudia Balotta, Giuseppe Calicchio

**Affiliations:** 1 Azienda Servizi alla Persona, Istituti Milanesi Martinitt e Stelline e Pio Albergo Trivulzio, Milan, Italy; 2 Infectious Diseases Unit, Legnano General Hospital, ASST Ovest Milanese, Legnano, Italy; 3 Department of Biomedical and Clinical Sciences L. Sacco, University of Milan, Via G.B. Grassi, 74, 20157 Milan, Italy; 4 Department of Medicine and Surgery, University of Milan-Bicocca, Monza, Italy; 5 Department of Biomedical Sciences, University of Milan and IRCCS Istituto Ortopedico Galeazzi, Milan, Italy

**Keywords:** SARS-CoV-2, Long term care facilities, Older age, Comorbidities, S-IgG antibodies

## Abstract

**Background:**

The impact of coronavirus disease 2019 (COVID-19) caused by Severe Acute Respiratory Syndrome Coronavirus 2 (SARS-CoV-2) on residents of long-term care facilities (LTCFs) has been dramatic on global scale as older age and comorbidities pose an increased risk of severe disease and death.

**Methods:**

Aim of this study was to evaluate SARS-CoV-2 Spike-specific IgG (S-IgG) antibody titers in 478 residents and 649 health care workers of a large Italian long-term care facility two months after complete vaccination with BNT162b2. Associations among resident-related factors and predictors of humoral response were investigated.

**Results:**

By stratifying levels of humoral responses, we found that 62.1%, 21.6%, 12.1% and 4.2% of residents had high (>1,000 BAU/ml), medium (101-1,000), low (1-100) and null (<1 BAU/mL) S-IgG titers, respectively. Residents with documented previous COVID-19 and those with SARS-CoV-2 nucleocapsid-specific IgG (N-IgG) positive serology showed higher level of serological response, while significant associations were observed for cancer with suboptimal response (*p = 0.005*) and the administration of corticosteroid for suboptimal response (*p = 0.028*) and a null one (*p = 0.039*). According to multivariate logistic regression, predictors of an increased risk of null response were advanced age (Odd ratio, OR: 2.630; Confidence interval, CI: 1.13-6.14; *p = 0.025*), corticosteroid therapy (OR: 4.964; CI: 1.06-23.52; *p = 0.042*) and diabetes mellitus (OR:3.415; CI:1.08-10.8; *p = 0.037).* In contrast, previous diagnosis of COVID-19 was strongly associated with a reduced risk of null response to vaccination (OR:0.126; CI:0.02-0.23; *p < 0.001*).

**Conclusions:**

SARS-CoV-2 specific antibodies in elderly individuals should be consider when deciding the need of a third dose of vaccine for prevention of reinfections in LTCFs despite the maintenance of barrier measures.

**Supplementary Information:**

The online version contains supplementary material available at 10.1186/s12877-022-02884-0.

## Introduction

The coronavirus disease 2019 (COVID-19) pandemic, which started in late 2019 in China, continues to spread worldwide, despite the adoption of personal protective equipment (PPE), subsequent lockdowns, prolonged control measures implemented in most developed countries and unprecedented vaccination campaigns. The impact of COVID-19 on older people living in long-term care facilities (LTCFs) has been particularly devastating at national and international scales [1]. Living in community, the lack of diagnostic tools as well as PPE for residents and health care workers (HCWs) and the health vulnerabilities of the elderly, all contributed to the spread and lethality of the virus in the setting of LTCFs. In addition, conditions like frailty, dependence, dementia or high burden of comorbidities are responsible of high incidence of disease susceptibility and mortality. By July 2021, the highest mortality rate among older people (aged >75 years), has reached 48.7% of confirmed cases as reported by New York City Health (Worldometer. Age, sex, existing conditions of COVID-19 cases and deaths, accessed July 8, 2021) (https://www.worldometers.info/coronavirus/coronavirus-age-sex-demographics/). In parallel, Italian data from the Istituto Superiore di Sanità (from February 2020 to May 2021) indicate that the overall mortality rate, defined as number of deaths over the total of residents, was 9.1% (https://www.epicentro.iss.it.coronavirus.pdf).

Age, concomitant pathologies and immunosenescence are recognized as the main factors that influence the risk of severe morbidity and death. Among these, it has been proven that deterioration of both humoral and cellular immune responses and alteration of lymph node architecture play a major role in the failure to contrast pathogens and related morbidity [2]. Indeed, immune responses may be affected both by aforementioned factors and other common events related to senescence, such as the alteration of metabolic processes, blood circulation, gas exchanges and organ function. Lower responses to influenza virus, *Streptococcus pneumoniae* and some Flaviviruses have been demonstrated in elderly compared to younger adults [3–8].

For these reasons elderly and residents of LTCF, as well as HCWs taking care of them, have been prioritized to be vaccinated as soon as vaccines became available (December 27, 2020).

Several reports indicate that vaccination of old age is showing to protect from severe disease, and risk of death by conferring a degree of humoral immunity [9–15].

Here, we present data obtained in the largest LTCF facility in Milan, the Italian city early hit by the SARS-CoV-2 pandemic. We demonstrate differences between elderly vaccinees over the age of 70 and young vaccinees below 60 years by investigating differences in antibody titers by age, and we identify factors related to humoral responses after the second dose of mRNA BNT162b2 vaccine. Implications of this study may be important to reinforce protective measures in NHs as well as to pose the need of to identify effective vaccination strategies in frail residents, including additional doses and/or switch to different vaccines.

## Methods

### Study population

The study was conducted at the Pio Albergo Trivulzio, the main long-term care facility (LTCF) in Milan, hosting about 500 aged residents and hiring about 700 HCW. Four hundred and seventy-eight LTCF residents and 649 HCWs were studied. HCWs included nurses, doctors, healthcare technicians, health service assistant, cleaners, laboratory and administrative staff.

First dose administration of BNT162b2 vaccine started in the structure on 27th December 2020 and occurred until 31th January 2021. Second dose was administered at 21 days and was completed on 25th February 2021.

Around two months after the second dose, a blood sample was obtained for routine assessment of laboratory parameters and a 500 µl were stored at -20° for SARS-CoV-2 antibody quantifications.

HCWs were administered vaccine and tested in parallel with informed consent.

Samples were concomitantly screened for SARS-CoV-2 nucleocapsid-specific IgG (N-IgG) to capture possible asymptomatic infection or assess the persistence of specific antibodies in previously infected subjects and SARS-CoV-2 Spike-specific IgG (S-IgG) antibodies. All data used in this study were previously anonymized as required by the Italian Data Protection Code (Legislative Decree 196/2003) and the general authorizations issued by the Data Protection Authority. Ethics Committee approval was deemed unnecessary because, under Italian law, it is only required in the case of prospective clinical trials of medical products for clinical use (Art. 6 and Art. 9 of Legislative Decree 211/2003). All participants provided written informed consent. If residents lacked the capacity to consent, a personal or nominated consultee was identified to act on their behalf. The study was conducted in compliance with Good Clinical Practice (https://ichgcp.net/it) and the declaration of Helsinky.

### SARS-CoV-2 antibody assays

We used Elecsys Anti-SARS-CoV-2 S (Roche), an immunoassay for the quantitative determination *in vitro* of total antibodies to the SARS-CoV-2 spike (S) protein receptor binding domain (RBD) in human serum and plasma. The assay cut off is >0.8 BAU/ml reported by manufacturer. Elecsys AntiSARSCoV2 S assay manufacturer reports a sensitivity of 9.8% (95% CI: 98.1–99.3%) and a specificity of95% (CI: 99.7–100%).

Response to vaccination in residents was classified as high, medium, low and null response by stratifying the level of anti-S IgG values in 4 levels: >1,000, 101-1,000, 1-100 and <1 BAU/mL, respectively.

Elecsys Anti-SARS-CoV-2 (Roche), an immunoassay for the in vitro quantitative determination of antibodies to the SARS-CoV-2 N protein in human serum and plasma was used with a sensitivity of 99.5% (CI: 97.0-100%) and a specificity of 99.80% (CI: 99.69-99.88).

### Statistical analysis

Descriptive analyses of demographic and clinical data are presented as median and Inter-Quartile Range (IQR) when continuous and as frequency and proportion (%) when categorical. Parametric tests (t test and ANOVA), nonparametric tests (Mann–Whitney and Kruskal–Wallis) and the Pearson χ2 test (or Fisher exact test, when necessary) were used to compare normally distributed, non-normally distributed continuous, and categorical variables of patients, respectively. The primary endpoint was the risk of null response to vaccine, evaluated by means of a logistic regression model, also correcting for gender, age, comorbidities and immune modulatory treatments. Also, previous diagnosis of COVID-19 and positive anti-nucleocapsid serology were included as correlates in the analysis in two different models, due to their strong reciprocal association. We evaluated their combined effect over the risk of null response to vaccination stratifying the population based on previous clinical and serological evidence of SARS-CoV-2 exposure. Significance was established at *p* <0.05. Data analysis was performed using the IBM SPSS Statistics version 25. Separated analyses were performed for LTCF residents and HCWs.

## Results

### Participant characteristics

#### Residents

The main demographic and clinical characteristics of 478 analyzed LTCF subjects are shown in Table 1. The majority of residents were female (81%) with an age higher than 80 years old (*n* = 345, 72.2%). SARS-CoV-2 anti-nucleocapsid antibodies determination was available for 455 subjects and resulted positive in 268 cases (58.9%). Among these, 143 residents (53.4%) did not show clinical signs of infection in the past, while 7 patients with documented clinical COVID-19 had a negative N-IgG result (7/187, 3.7%).

**Table 1:** Characteristics of 478 subjects undergoing to SARS-CoV-2 vaccination in a long-term care facility. Categorical variables are expressed as % (n), continuous variables as median value (Inter-quartile range, IQR)

		% (n)
Gender, female		81.0 (387)
Age, years (IQR)		87 (82-92)
Age distribution		
<70 years		5.0 (24)
70-80 years		11.1 (53)
80-90 years		47.5 (227)
>90 years		36.4 (174)
Documented clinical COVID-19 infection		29.7 (142)
Diabetes		15.5 (74)
Cancer		4.8 (23)
Malnutrition		15.5 (74)
Heart Disease		4.0 (19)
COPD^a^		13.4 (64)
Cerebral stroke		15.7 (75)
Dementia		58.6 (280)
Autoimmune disease		1.0 (5)
Gastrointestinal/Liver Disease		1.3 (6)
Chronic kidney disease		1.7 (8)
Anemia		5.6 (27)
Immunosuppressive therapy		0.6 (3)
Anticoagulant therapy		23.8 (114)
O2 therapy		4.8 (23)
Corticosteroid therapy		4.0 (19)
Previous positive anti-nucleocapsid serology		58.9 (268/455)
Anti-spike antibodies median titre (IQR)		>7,500 (848-7,500)

^a^Chronic obstructive pulmonary disease

A large majority (*n* = 413, 86.4%) of residents presented at least one morbidity, being affected by dementia, the most common syndrome, in 58.6% of cases. Almost all residents received polytherapies for chronic diseases (hypertension, heart disease, lung disease, diabetes and cancer). Anticoagulant or corticosteroid therapy were administered in 114/478 (23.8%) and 19/478 (4.0%), respectively.

### HCWs

Among the 649 HCWs, the majority of subjects were females (*n* = 464, 71.5%) with a median age of 49 years (IQR: 34-55). In detail, the age classes were as follows: 15.1% (*n* = 98), 18.2% (*n* = 118), 21.6% (*n* = 140), 36.8% (*n* = 239) and 8.3% (*n* = 54) for ≥30, 31-40, 41-50, 51-60 and >60 years, respectively. About one third of subjects (27.3%, *n* = 113) presented at least one comorbidity, while 14 presented two of them. HCWs with a previous exposure to SARS-CoV-2 were 124 (19.1%) as detected by nasopharyngeal swab (data not shown).

### Vaccine-associated side effects

No major vaccine associated side effects occurred either in residents or in HCWs. Minor effects such as injection site pain, fatigue, malaise, headache, nausea or skin rash, muscle and joint pain, fever were reported in about 10% and 16% of individuals, respectively.

### Serological response to vaccine

#### Residents

Overall, we detected anti-S-IgG antibodies in 95.8% of residents receiving two doses of BNT162b2 vaccine. According to level of response, high, medium and low titers were present in 62.1% (*n* = 297), 21.6% (*n* = 103) and 12.1% (*n* = 58) of cases, respectively.

Median level of anti-S IgG of 7,500, corresponding to the upper limit of quantification of the assay (IQR: 848 - >7,500 BAU/mL) after a median time of 64 days from the second dose (IQR 63-65 days).

We first investigated the associations among main epidemiological and clinical characteristics and levels of response to vaccination. The results are reported in Table 2.

**Table 2:** Response to SARS-CoV-2 vaccination among 478 patients living in a long-term care facility, according to the main epidemiological and clinical characteristics showing significant associations and differences among groups. Pearson χ2 test was used to compare categorical variables of patients in the study groups

		Grade of response to SARS-CoV-2 vaccine, % (n)				Comparison of response distribution, ***p***-value		
		High response ***n*** = 297	Medium response ***n*** = 103	Low response ***n*** = 58	Null response ***n*** = 20	Overall*	Suboptimal response**	Null response***
Gender	Females	61.7 (239)	22.5 (87)	10.9 (42)	4.9 (19)	ns	ns	0.050
	Males	63.7 (58)	17.6 (16)	17.6 (16)	1.1 (1)			
Age groups, years	<70	58.3 (14)	29.2 (7)	8.3 (2)	4.2 (1)	ns	ns	0.061
	70-80	62.3 (33)	20.8 (11)	113.2 (7)	3.8 (2)			
	80-90	63.4 (144)	50.0 (22)	11.9 (27)	2.6 (6)			
	>90	60.9 (106)	20.1 (35)	12.6 (22)	6.3 (11)			
Documented clinical COVID-19 infection	No	49.1 (165)	28.9 (97)	16.1 (54)	5.9 (20)	<0.001	<0.001	0.005
	Yes	92.9 (132)	4.2 (6)	2.8 (4)	0.0 (0)			
Anti-Nucleocapsid serology	Neg	17.1 (32)	47.6 (89)	25.1 (47)	10.2 (19)	<0.001	<0.001	<0.001
	Pos	98.1 (263)	1.1 (3)	0.4 (1)	0.4 (1)			
Diabetes	No	64.1 (259)	20.9 (85)	12.0 (48)	3.0 (12)	0.077	ns	0.052
	Yes	51.9 (38)	24.7 (18)	13.0 (10)	10.4 (8)			
Cancer	No	63.9 (291)	21.1 (96)	11.0 (50)	4.0 (18)	0.004	0.005	ns
	Yes	29.2 (7)	29.2 (7)	33.3 (8)	8.3 (2)			
Corticosteroid therapy	No	63.2 (290)	21.3 (98)	12.0 (55)	3.5 (16)	0.019	0.028	0.039
	Yes	36.8 (7)	26.3 (5)	15.8 (3)	21.0 (4)			

* Overall *p*-value: comparison between all the response groups

** Suboptimal response *p*-value: comparison of low-null response vs. medium-high response

*** Null response *p*-value: comparison of null response vs. all the other responses grouped together

A weak association for null response was observed for female compared to male sex (4.9% vs. 1.1%, *p = 0.050*) and diabetes (10.4% vs. 3% *p = 0.052*). A significantly higher response was detected for residents with previous COVID-19 and for those with SARS-CoV-2 N-IgG serology considering all level of serological response (*p < 0.001* and *p < 0.001*). In contrast, the administration of corticosteroid diminished all levels of specific antibodies (*p = 0.019*). Significant associations were observed for these parameters in those with suboptimal response (*p < 0.001*, *p < 0.001 and p = 0.028*) and with a null one (*p = 0.005, p < 0.001* and *p = 0.039*).

Among subjects with a previous COVID-19 clinical diagnosis, we did not find any case of null response to vaccine, either in those with positive or negative nucleocapisd serology (0/125 and 0/7, respectively). Differently, among residents without a documented previous diagnosis of COVID-19, null response to vaccine was lower in those with positive nucleocapisd serology when compared to subjects with negative serology: 0.7% (1/143) vs. 10.6% (19/180), respectively (*p < 0.001*) (data not shown).

LTCF residents with neoplastic disease showed a significant difference in the distribution of antibody response considering all levels (*p = 0.004*) and suboptimal response (*p = 0.005*) but not null response.

### HCWs

Regarding the response to vaccination, 66.1% (*n* = 429) of subjects showed high titers and 33% (*n* = 214) medium titers. A suboptimal response was observed in 6 subjects (0.9%), of whom 2 were null responder. The median level of anti-S IgG in HCW was 1,789 (IQR: 754->7,500), markedly lower compared to residents, according to the lower percentage of previously infection in these subjects. A significantly different distribution of response was detected for age classes both considering all titer strata (*p < 0.001*) and null response (*p = 0.040*). Null responders were present only in subjects with age of more than 60 years (*n* = 2, 3.7%). A significantly different distribution of response was present for previous COVID-19 only considering all strata of antibody titers (*p < 0.001*), as the only two non-responders did not experience COVID-19 (data not shown).

### Predictors of responses in LTCF residents and HCWs

We then studied predictors of null response to SARS-CoV-2 vaccination by univariate and multivariate logistic regression analysis (Table 3). Firstly, the advanced age was strongly associated with an increased risk of null response (Odd ratio, OR: 2.988; Confidence interval, CI: 1.40-6.39; and OR: 2.630; CI: 1.13-6.14; in univariate and multivariate analysis, respectively). In addition, corticosteroid therapy was associated with no anti-spike antibody titers in both analyses (OR: 3.246; CI: 1.23-8.54; and OR: 4.964; CI: 1.06-23.52). Diabetes mellitus was significantly associated with a higher risk of null response only in the multivariate analysis (OR:3.415; CI:1.08-10.8).

**Table 3:** Logistic regression model evaluating the risk of null response to SARS-CoV-2 vaccination among 478 patients living in a long-term care facility

	Univariate analysis			Multivariate analysis		
	*p*	OR^a^	95% CI^b^	*f*	OR	95% CI
Age, per 10 year higher	0.005	2.988	1.40-6.39	0.025	2.630	1.13-6.14
Gender, females vs. males	0.123	4.905	0.65-37.0	0.292	3.103	0.38-25.50
Documented clinical COVID-19 infection 9	0.000	0.103	0.01-0.12	0.000	0.126	0.02-0.23
Diabetes mellitus	0.060	2.613	0.96-7.11	0.037	3.415	1.08-10.8
Cancer	0.936	1.088	0.14-8.53	0.377	0.357	0.04-3.51
Corticosteroid therapy	0.017	3.246	1.23-8.54	0.042	4.964	1.06-23.52

^a^ Confidence interval

^b^ Odd ratio

A previous diagnosis of COVID-19 was strongly associated with a reduced risk of null response to vaccination (OR:0.126; CI:0.02-0.23). No association was observed for cancer and female sex either in the univariate or multivariate analyses.

When evaluating positive anti-nucleocapsid serology instead of COVID-19 clinical diagnosis, the former was associated with a lower risk of null response to vaccine, both in the univariate (OR: 0.035, CI: 0.01-0.26) and in the multivariate analysis (OR: 0.051, CI: 0.01-0.42), with no impact on significance of other covariates (data not shown).

Univariate and multivariate analyses for HCW-related variables did not show any factor is associated with a null response to vaccination (data not shown).

## Discussion

Serosurveys are of great importance to define the serological response to the COVID-19 vaccine in a real world population, particularly in fragile individuals. In general, quantification of the antibody response to SARS-CoV-2 vaccination is highly relevant for identifying possible vaccine failure (i.e. the risk of breakthrough infection) and estimating level and time of protection. However, thresholds for positivity and cut off values provided by different assay manufacturers differ and their diagnostic value is not yet established and standardized at present. Although total anti-spike titers may be not indicative of sufficient inhibitory capacities, vaccination-induced antibody titers may be used as surrogate marker from which a protection correlate could be estimated [16, 17]. Recent papers addressed the evaluation of vaccine responses after the first dose in elderly suggesting that anti-S antibody levels are markedly influenced by previous infection and may be delayed [18, 19]. Moreover, Collier et al. indicated that age-related immune responses may be heterogeneous [20].

Several papers addressed the efficacy of different SARS-CoV-2 vaccines either through Phase II and III of clinical trials [21–23] or field investigations [9–11]. Few of them detailed data regarding elderly and persons living in LTCF [12–15] and outbreaks after the first and second dose were reported [24–26]. At present, cohorts <16 years or >80 years who might show reduced vaccine reactiveness are limited. We report a large sero-survey in residents of the largest LTCF of Italy where SARS-CoV-2 could spread because of late warning and lack of PPE and diagnostic tools. Our data indicate that full vaccination either in LTCF residents and HCWs elicits a humoral response in above 96% of individuals accordingly with published papers. Nevertheless, while the majority of elderly vaccinees and HCWs raised high responses after their second vaccination dose, a high percentage of residents showed a lower or null response when compared to HCW (14.2% vs. 0.9%). The main differences between the two groups of vaccinated individuals are likely a consequence of immunosenescence, which describes the phenomenon of reduced adaptive immune responses in residents. Previous data reported that titers of S-IgG antibodies are significantly lower in elderly persons [21]. Accordingly, our regression analysis in LTCF residents indicates that older age is strongly associated with an increased risk of null response.

Noteworthy, our findings suggest that comorbidities and their treatment may impact humoral responses as detected by several significant associations, even though only diabetes and corticosteroid therapy confer an increased risk of unresponsiveness. Nevertheless, corticosteroid therapy has been observed in a limited number of LTCF residents, suggesting that our observation should be interpreted with caution and confirmed in other elderly cohorts. Moreover, the low frequencies of other morbidities such as cancer and diseases of the immune system prevent us from evaluating other associations impacting antibody response. To our knowledge no similar data are yet available in elderly persons.

Our study population includes older adults living in assisted structures and their care providers, with or without a history of natural infection with SARS-CoV-2. As already reported, individuals having a history of natural infection or positive serology have higher antibody levels and an enhanced response to vaccination, even after a single dose [27]. Indeed, in our case-file both prevalence of diagnosed COVID-19 and median titers of S-IgG were higher in residents compared to HCWs (56% vs. 19.1% and 7,500 vs. 1,789 BAU/mL, respectively). Consequently, both N-IgG positive results and previous COVID-19 were predictive factors of favourable response to vaccination.

Although serological assays still need to be standardized, compared and interpreted in the light of large population results, they allow to preliminarily evaluate levels of titers conferred by SARS-CoV-2 vaccines. As demonstrated for other vaccine campaigns, vaccination of older individuals often fails to induce high titers of antibodies or fully protective immunity as quality and quantity of antibody titers may be markedly inferior in elderly compared to adults [2–8, 13, 28].

The major limitation of our study is that we measured spike (S) protein RBD antibodies, stratifying the level of response in high, medium, inadequate and null instead of evaluating neutralizing antibodies that are considered the better correlate of protection. Further differences in humoral response may be underscored in our study as a high percentage of values are above the upper limit of the assay quantification. We could not address this point by dilution experiments because of the large number of studied subjects. In addition, we measured S-IgG antibodies around two months after the vaccine administration. It is conceivable that antibodies titers could be above the ELISA dynamic range. Future studies to monitor antibody levels will clarify the dynamic of antibody mount and decay.

Our considerations are based on the hypothesis anti-SARS-CoV-2 antibodies are the major correlate of protection. Indeed, immunity is a complex phenomenon where both humoral and cellular responses are interdependent involving both innate and adaptive immunity. Further studies will be essential to understand type and function of antibodies produced after vaccination, the neutralizing capacity of the antibodies along with the persistence of their protective effects. Therefore, information regarding S-IgG levels are as much crucial as the identification of SARS-CoV-2 emerging variants that may elude protection conferred by vaccines. Although the effectiveness of mRNA vaccines may be inferred by screening methods based on sources of population-based data, further studies into how results from standardised assays can be used at an individual level to determine the degree of protection from SARS-CoV-2 in elderly individual are essential [29].

Elderly subjects who have been heavily affected by the SARS-CoV-2 pandemic need to be protected through general prevention actions and specific measures that include early vaccine administration and testing of their effects on humoral responses. As demonstrated in individuals with solid organ transplantation [30], a third booster dose of COVID-19 vaccine should be strongly considered in geriatric subpopulations at higher risk of not responding to the vaccine.

## Supplementary Information

**Additional file 1.**

## Data Availability

All data generated or analysed during this study are included in this published article and its supplementary information files.

## Abbreviations

SARS-CoV-2: Severe Acute Respiratory Syndrome Coronavirus 2
LTCFs: Long Term Care Facilities
COVID-19: Coronavirus disease 2019
PPE: Protective Equipment
HCWs: Health Care Workers
S: Spike protein
RBD: Receptor Binding Domain
N: Nucleocapisd protein
IQR: Inter-Quartile Range
COPD: Chronic Obstructive Pulmonary Disease
CI: Confidence Interval
OR: Odd Ratio
